# Electroacupuncture for patients with irritable bowel syndrome: A systematic review and meta-analysis protocol

**DOI:** 10.1097/MD.0000000000011627

**Published:** 2018-08-03

**Authors:** Yu Zhang, Ziqing Li, Fan Han

**Affiliations:** a The Second Clinical School of Guangzhou University of Chinese Medicine; b The Second Affiliated Hospital of Guangzhou University of Chinese Medicine, Guangzhou, China.

**Keywords:** electroacupuncture, irritable bowel syndrome, protocol, systematic review

## Abstract

**Background:**

Irritable bowel syndrome (IBS) is a common functional intestinal disease characterized by chronic or recurrent abdominal pain, abdominal distension, constipation and diarrhea. Many IBS sufferers are usually present with poor quality of life for the abdominal discomfort, diarrhea, constipation, and other complaints. At present, the commonly used drug therapy for IBS in the Western clinic, such as antidiarrheal, laxative, gastrointestinal antispasmodic, often cannot get satisfying curative effect. Thus complementary therapies like electroacupuncture (EA) which may be effective to decrease patients’ pain and fewer side-effects will be sought. EA, an innovative form of traditional acupuncture, is drawing more attention to the treatment of IBS due to its roles in symptom improvement. This systematic review protocol aims at describing a meta-analysis to critically evaluate the effectiveness and safety on EA for patients with IBS.

**Methods:**

We search Ovid MEDLINE, Ovid Embase, PubMed, the Cochrane Central Register of Controlled Trials (Cochrane Library), the Allied and Complementary Medicine Databases (AMED), China National Knowledge Infrastructure (CNKI) for random controlled trials EA for IBS from their inception to April 1, 2018. Two reviewers will independently screen studies for data extraction and assess the quality and risk of bias. RevManV5. 3 will be applied to data extraction. Risk of bias for each RCT will be assessed according to criteria by the Cochrane Handbook to evaluate the methodological quality.

**Results:**

This study will provide a high-quality synthesis of current evidence of effectiveness and safety on EA for patients with IBS.

**Conclusion:**

The conclusion of our systematic review will provide evidence to judge whether EA is an effective intervention for patient with IBS.

**PROSPERO registration number:**

PROSPERO CRD42018081610.

## Abstract 2

Supplemental Digital Content is available in the text

## 1 Introduction

Irritable bowel syndrome (IBS) is a common functional intestinal disease characterized by chronic or recurrent abdominal pain or abdominal discomfort, stool irregularities with high population prevalence.^[1]^ A study has reported that 6%–11. 5% of the population in various Asian countries has affected IBS.^[2]^ Among IBS sufferers, the prevalence of constipation-predominant in women is 38%, which is higher than male patients.^[3]^ IBS mostly occurs in the people aged between 30 and 50 years.^[4]^ The symptoms of IBS will bring the influences of depression, anxiety and somatoform disorder, which will reduce the quality of life significantly.^[5]^ Furthermore, the tremendous economic burden associated with IBS that brings to individuals, families, and society cannot be neglected. In the United State, the total direct costs associated with per IBS patient in 2013 have been estimated at $6, 182 per year.^[6]^

Effective treatments for IBS are considered to alleviate symptoms, ease economic burden, and enhance the quality of life. The traditional therapeutic drugs for IBS include antidiarrheal, laxative, gastrointestinal antispasmodic, antidepressant, and intestinal flora regulation. However, all of these drugs cannot obtain satisfactory curative effect and have significant limitations and side effects. With the continuous study of IBS pathogenesis, many potential therapeutic targets and drugs have been developed, including 5-HT antagonists, chloride channel, intestinal immune regulator. According to a study published by the American College of Gastroenterology Task Force, they affirmed the “good quality of evidence” for 5-HT antagonists, but supplemented that these drugs could increase the risk of ischemic colitis and cardiovascular events, which may restrict the application.^[7]^ The specific probiotic B. infantis 35624, one of the intestinal microflora, has been corroborated its effectiveness in the treatment of IBS.^[8]^ The results of these studies demonstrate that current situation of therapeutic drugs in IBS is not optimistic. Thus, there is an unmet need for new therapies for the treatment of IBS.

Due to the unsatisfactory result of conventional drug treatment of IBS, the IBS patients pay more attention to complementary and alternative medicine(CAM) modality.^[9]^ Acupuncture, an important part of CAM, has been shown its effectiveness in the treatment of various pain and gastrointestinal disorders, which includes IBS.^[10,11]^ EA is an advanced version of traditional acupuncture that inserts needles and provides stimulation with the application of electrical pulses instead of hand manipulation, which has promising treatment effect. Several trials have demonstrated EA is effective for the treatment of IBS.^[12–17]^ EA is commonly used in China to treat functional bowel disorders such as IBS. Since IBS is diagnosed based on its clinical symptoms and its pathophysiology is unclear, the mechanism that EA is effective against IBS has not yet been completely ascertained and needs to be further studied. One animal study demonstrated that EA achieves remission of the IBS visceral hypersensitivity by regulating the expression of purinergic receptors in the peripheral and central pathways to improve in gastrointestinal symptoms.^[18]^ In another study, EA at PC 6 was ascertained to have significant reduction to the frequency of transient lower esophageal relaxations (TLESRs) caused by gastric distension in normal cats.^[19]^ In dogs with intestinal motility observed by duplex Doppler sonography, EA at BL27 was reported to decrease the frequency of intestinal movement by 31%.^[19]^

Although managing IBS with EA is common in China, there is still a lack of systematic review to summarize the efficiency of EA in treating IBS. Therefore, it is necessary for this review to investigate the role of EA in the management of IBS. Several randomized controlled trials (RCTs) have been published which indicated the benefits of EA in treating IBS. Apparently, the update of systematic review and meta-analysis of EA for IBS is an unmet need to evaluate whether the pooled effects of currently available trials show any benefit of EA in improving symptoms or quality of life in patients with IBS.

## 2 Methods

### 2.1 Inclusion criteria for study selection

#### 2.1.1 Types of studies

All RCTs comparing no interventions, sham acupuncture, placebo control for the treatment of IBS with EA treatment that are published as a full text in a peer-reviewed journal, will be eligible for inclusion in the review, while cross-over studies will be excluded. It is impossible to blind the acupuncturists for the particularity of EA manipulation. Hence, blinding will not be considered into the inclusion criteria.

#### 2.1.2 Type of participants

Patients who are diagnosed with IBS according to Manning or Rome I, II, or III criteria will be included in the analysis, regardless of their age, gender, ethnicity, or background.

#### 2.1.3 Type of interventions

In accordance with earlier Cochrane reviews, only the EA interventions for the treatment of IBS will be considered in experimental groups. The control group will be treated with no intervention, sham acupuncture, and placebo control.

#### 2.1.4 Type of outcome measures

The post-treatment IBS symptom severity scores and quality of life measures in IBS will be considered 2 of the most appropriate IBS symptom outcome measures. The primary outcome will be the post-treatment IBS symptom severity scores, while the secondary outcome measures will be the improvement of symptoms of anxiety and depression as well as quality of life. Studies without relevant information about the efficacy of the EA interventions will be excluded. We will extract outcome data from the studies that report the proportion of participants in each group with overall symptom improvement after treatment.

### 2.2 Search methods for identification of studies

#### 2.2.1 Electronic searches

To ascertain the relevant information, the following databases, Ovid MEDLINE, Ovid Embase, PubMed, the Cochrane Central Register of Controlled Trials (Cochrane Library), the Allied and Complementary Medicine Databases (AMED), and China National Knowledge Infrastructure (CNKI), will be utilized for conducting electronic searches. The search strategy for PubMed will be exhibited in Appendix A.

#### 2.2.2 Searching other resources

Potential eligible studies will be searched through relevant conference proceedings and reference list of previously published reviews.

### 2.3 Data collection and analysis

#### 2.3.1 Selection of studies

**Figure 1:**
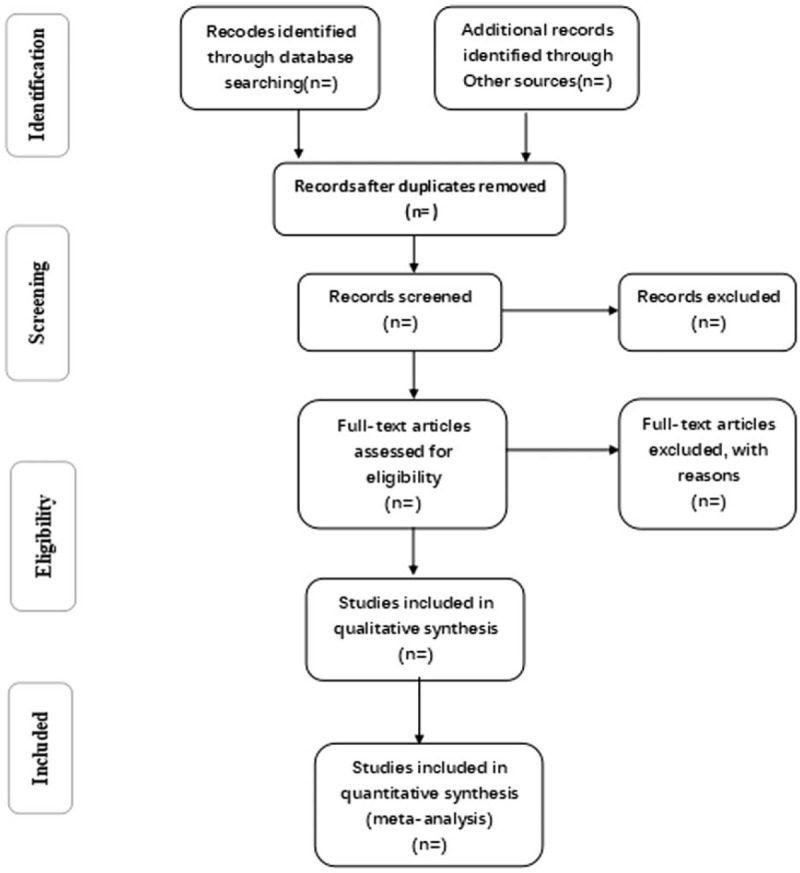
Flow diagram of study selection process.

Finishing the initial database search, duplicate literature will be eliminated and the eligible studies searched from above-mentioned databases will be transferred a database set up by EndNotesX7. Two reviewers will review the full text of these studies to ascertain their eligibility according to a standardized eligibility criteria sheet. The detailed reasons for elimination of trials will be registered when screening all papers. Any disagreement during the course will be resolved by discussion between the 2 reviewers. If a consensus cannot be achieved, an independent reviewer will be consulted. Details of the selection procedure for studies are shown in a PRISMA flowchart (Fig. 1).

#### 2.3.2 Data extraction and management

From the final selection of papers, general study information on the number of patients, patient characteristics (gender, mean age, and mean duration of illness), criteria for diagnosis (Rome I, Rome II, Rome III, or Manning), treatment setting, intervention (group or individual delivery format, type, number of sessions, training of therapists and use of treatment/ placebo manual), placebo control (type, group or individual delivery format, number of sessions, training of therapists and use of treatment/placebo manual), duration of the follow-up period, duration of treatment, and results relating to the primary and secondary outcome measures will be extracted.

#### 2.3.3 Assessment of risk of bias in included studies

The risk of bias in each included study will be assessed independently by 2 review authors utilizing the Cochrane Collaboration's risk of bias tool, which consists of the following 7 domains that may bring the potential risks of overestimating or underestimating an intervention effect: sequence generation, blinding of participants, blinding of outcome assessors, allocation concealment, incomplete outcome data, selective outcome reporting, and other sources of bias. The assessment will be ranked risk level within each category as low risk, high risk, and unclear risk.

#### 2.3.4 Measures of treatment effect

For dichotomous data, the risk ratio (RR) with 95% CIs will be utilized for analysis. For continuous outcomes, the mean difference (MD) or standard MD (SMD) will be utilized for evaluating the treatment effect with 95% CIs.

#### 2.3.5 Unit of analysis issue

Only the first experimental period data will be assessed in randomized cross-over trials to obviate carry-out effects. For all studies with multiple intervention groups in common, we will implement pairwise comparison if the groups satisfy the predefined inclusion standard. Only RCTs will be considered for analysis.

#### 2.3.6 Dealing with missing data

When the trial data is missing or insufficient, we will attempt to contact the original authors of the considered studies by e-mail or by telephone for adequate and comprehensive data. If we fail to retrieve inadequate data, the unavailable data will be abandoned. And the potential impacts of the missing data will be addressed in the following discussion.

#### 2.3.7 Assessment of heterogeneity

The *I*^2^ statistic will be utilized for assessing the heterogeneity of the included trials. We consider that the study has significant statistic heterogeneity if the I^2^ value of 50% or more. When the *I*^2^ value is no >50%, the study will not be considered to be an indicator of substantial level of heterogeneity. Subgroup analysis will be carried out to ascertain possible reasons.

#### 2.3.8 Assessment of reporting biases

We will utilize visual asymmetry on a funnel plot to detect reporting biases and small-study effects using adequate numbers of considered studies (>9 studies).

#### 2.3.9 Data synthesis

When meta-analysis is available, RevManV5.3 will be applied to analyze data. Data will use a random effects model with 95% CIs as substantial heterogeneity is expected among included studies. If the *I*^2^ test is >75%, we will not perform meta-analysis if the heterogeneity cannot ascertain possible causes from both clinical and methodological diversity. The fixed-effects model will be utilized for data synthesis if the *I*^2^ is <50%, while the random-effects model will be performed for data synthesis when the *I*^2^ is in the range of 50% to 75%.

#### 2.3.10 Subgroup analysis

If the number of available studies is sufficient enough (at least 10 trials), the conduction of subgroups analysis will be taken into account to explore the possible reason of insignificant heterogeneity. If heterogeneity is significant, we will also perform subgroups analysis when necessary.

#### 2.3.11 Sensitivity analysis

Sensitivity analysis will be conducted to determine the robustness of the results in the review. The principal criteria include simple size, methodological quality and the effect of the missing data. The meta-analysis will be performed repeatedly and trials of lower quality will not be included.

#### 2.3.12 Grading the quality of evidence

The Grading of Recommendations Assessment, Development and Evaluation (GRADE) will be utilized for assessing the quality of evidence for the main outcomes. The quality of evidence will be categorized into 4 levels: high, moderate, low, and very low quality.

#### 2.3.13 Ethics and dissemination

This systematic review will not use data from individual patients to protect privacy, and the results of this systematic review will be disseminated only in a peer reviewed publication.

## 3 Discussion

IBS is unclear for its structural or biochemical abnormalities but often associated with other somatic comorbidities (e.g., pain syndromes, overactive bladder and migraine), psychiatric conditions (including depression and anxiety) and visceral sensitivity.^[20]^

However, the conventional therapeutic options cannot satisfy the expected therapeutic effect and bring unavoidable side-effects.^[21,22]^ Recently, EA has appealed to some scholars and IBS sufferers.

EA is a complementary and alternative medicine which is commonly used as an adjuvant therapy in many clinical conditions to reduce adverse drug reaction and improve treatment. Studies have confirmed the effectiveness of acupuncture in relieving the symptoms of IBS.^[23]^ Nevertheless, to best of our knowledge, there is no systematic review related to EA for IBS published in English. Therefore, we conduct this systematic review to further study the effectiveness of EA in treating IBS. However, this study exists inevitable risk that sham acupuncture control is sufficiently believable to IBS patients as to be indistinguishable from true acupuncture, which may run risk of heterogeneity. Besides, we only selected the included trials in English or Chinese, which may cause potential studies losing.

## Author contributions

**Conceptualization:** Fan Han, Ziqing Li, Yu Zhang

**Data curation:** Ziqing Li, Yu Zhang.

**Formal analysis:** Fan Han, Yu Zhang.

**Funding acquisition:** Fan Han.

## Supplementary Material

Supplemental Digital Content
