# Supplementary material for: Electroacupuncture for patients with irritable bowel syndrome: A systematic review and meta-analysis protocol
Source: Medicine (Baltimore). 2018 Aug 3;97(31):e11627. doi: 10.1097/MD.0000000000011627 (PMC6081165; doi:10.1097/MD.0000000000011627)
Supplement: Supplemental Digital Content [file medi-97-e11627-s001.docx]

**Appendix A.**

**Search strategy used in PubMed database**

#1 Electroacupuncture

#2 Irritable Bowel Syndromes OR Syndrome, Irritable Bowel OR Syndromes, Irritable Bowel OR Colon, Irritable OR Irritable Colon OR Colitis, Mucous OR Colitides, Mucous OR Mucous Colitides OR Mucous Colitis

#3 Randomized controlled trial OR clinical study OR Clin-ical Trial OR Controlled study OR Controlled Trial OR Random*Control* study OR random* Control* Trial

#1 AND #2 AND #3
